# Supplementary figures and images for: Calaxin stabilizes the docking of outer arm dyneins onto ciliary doublet microtubule in vertebrates
Source: eLife. 2023 Apr 14;12:e84860. doi: 10.7554/eLife.84860 (PMC10139691; doi:10.7554/eLife.84860)

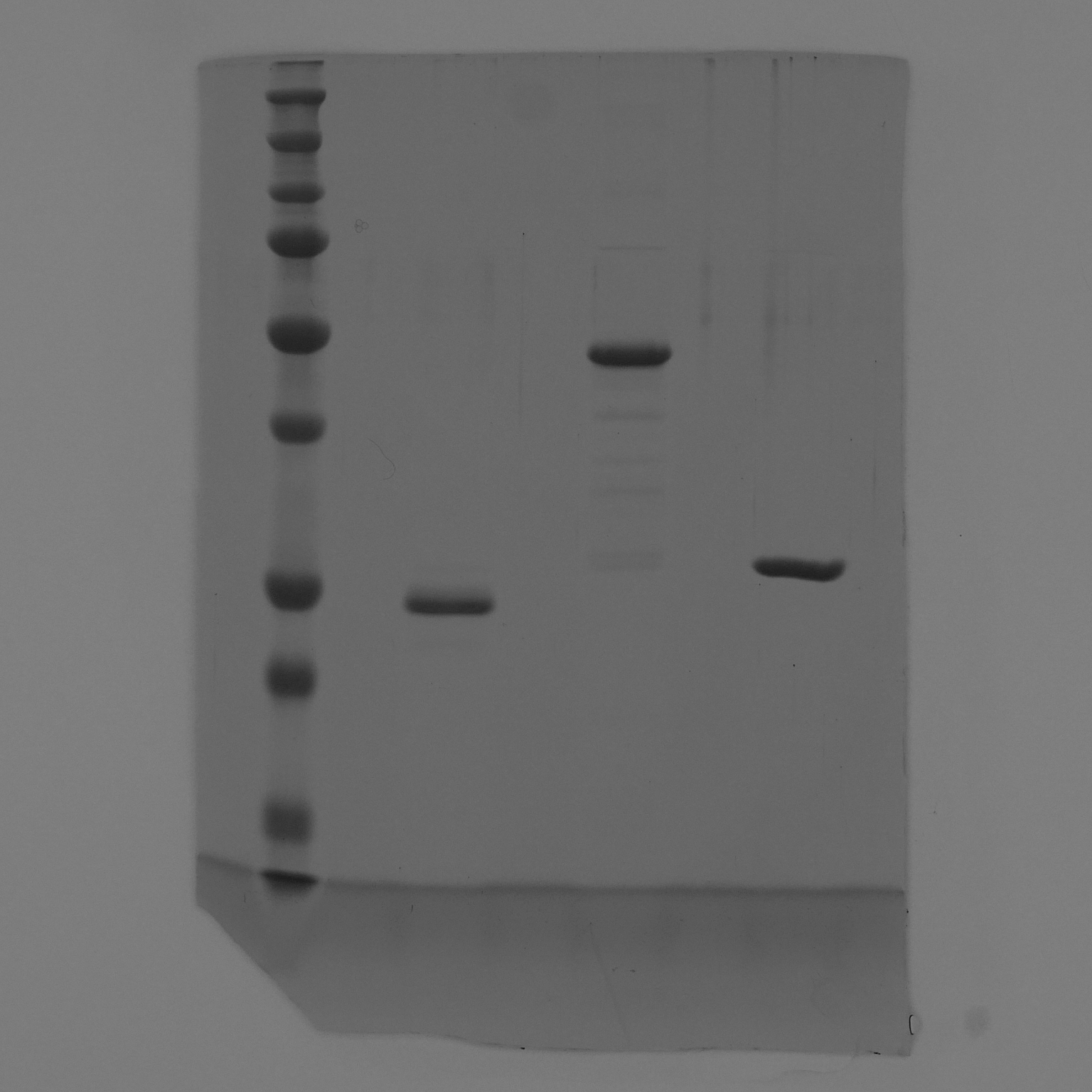

Supplement: Figure 4—source data 1. [file elife-84860-fig4-data1.zip › Figure 4-source data 1/Figure 4-source data 1_SDS-PAGE.jpg]

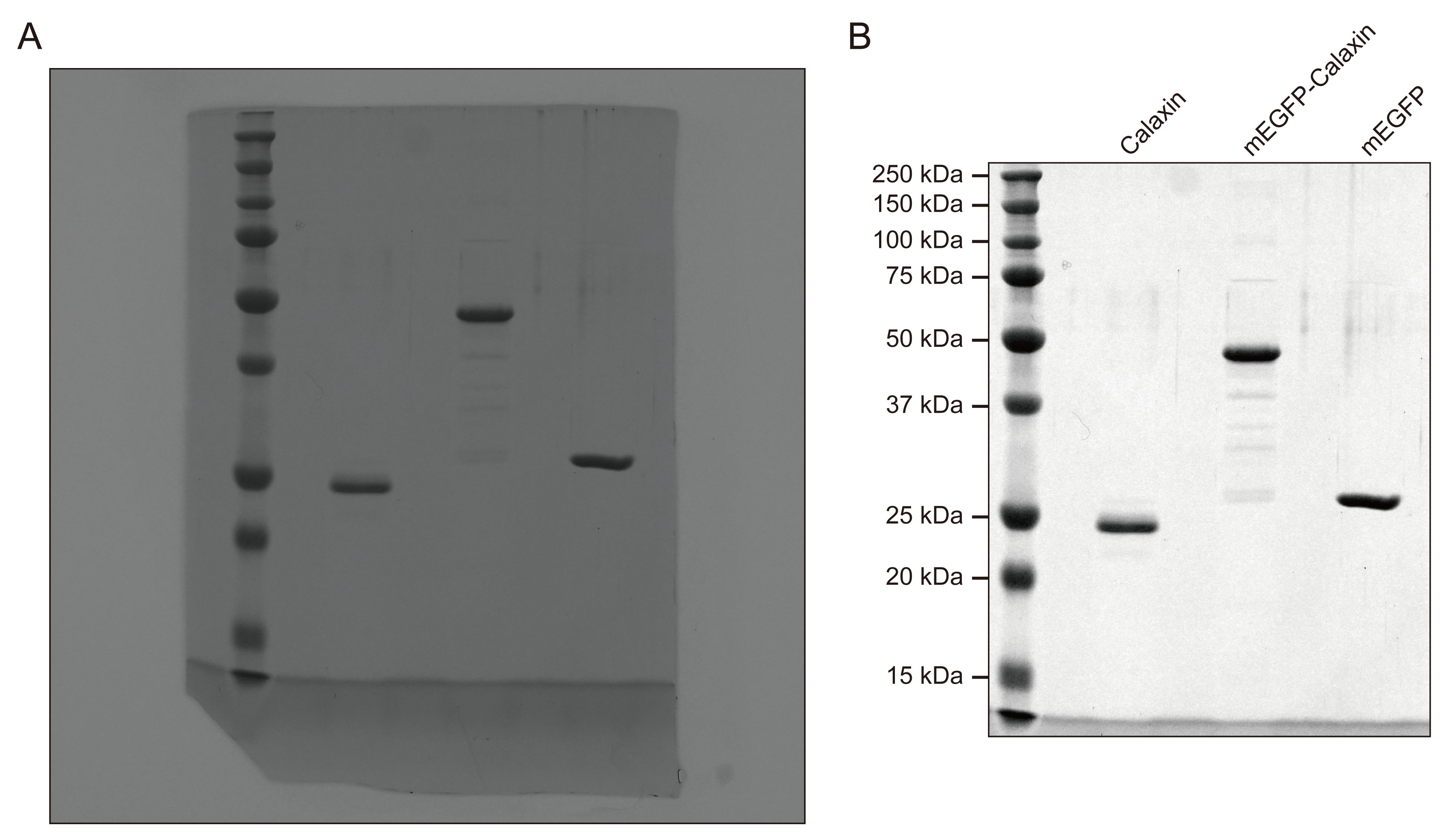

Supplement: Figure 4—source data 2. — (A) Original SDS-PAGE image. (B) Contrast adjusted image of A, with annotations for each lane. [file elife-84860-fig4-data2.zip › Figure 4-source data 2/Figure 4-source data 2.jpg]

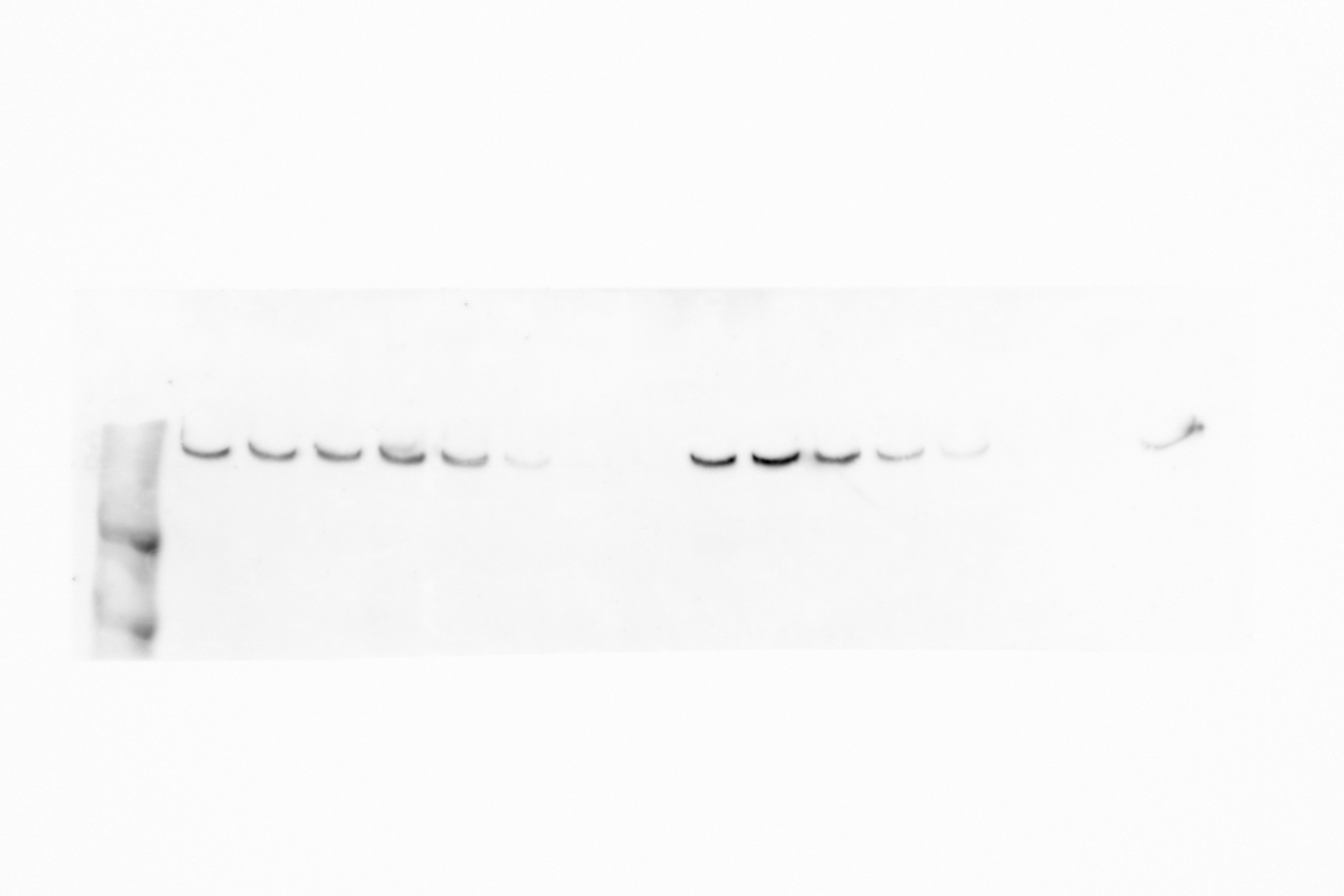

Supplement: Figure 5—source data 1. — Chemiluminescence and epi-illumination images of the blot membrane. [file elife-84860-fig5-data1.zip › Figure 5-source data 1/Figure 5-source data 1_chemiluminescence.tif]

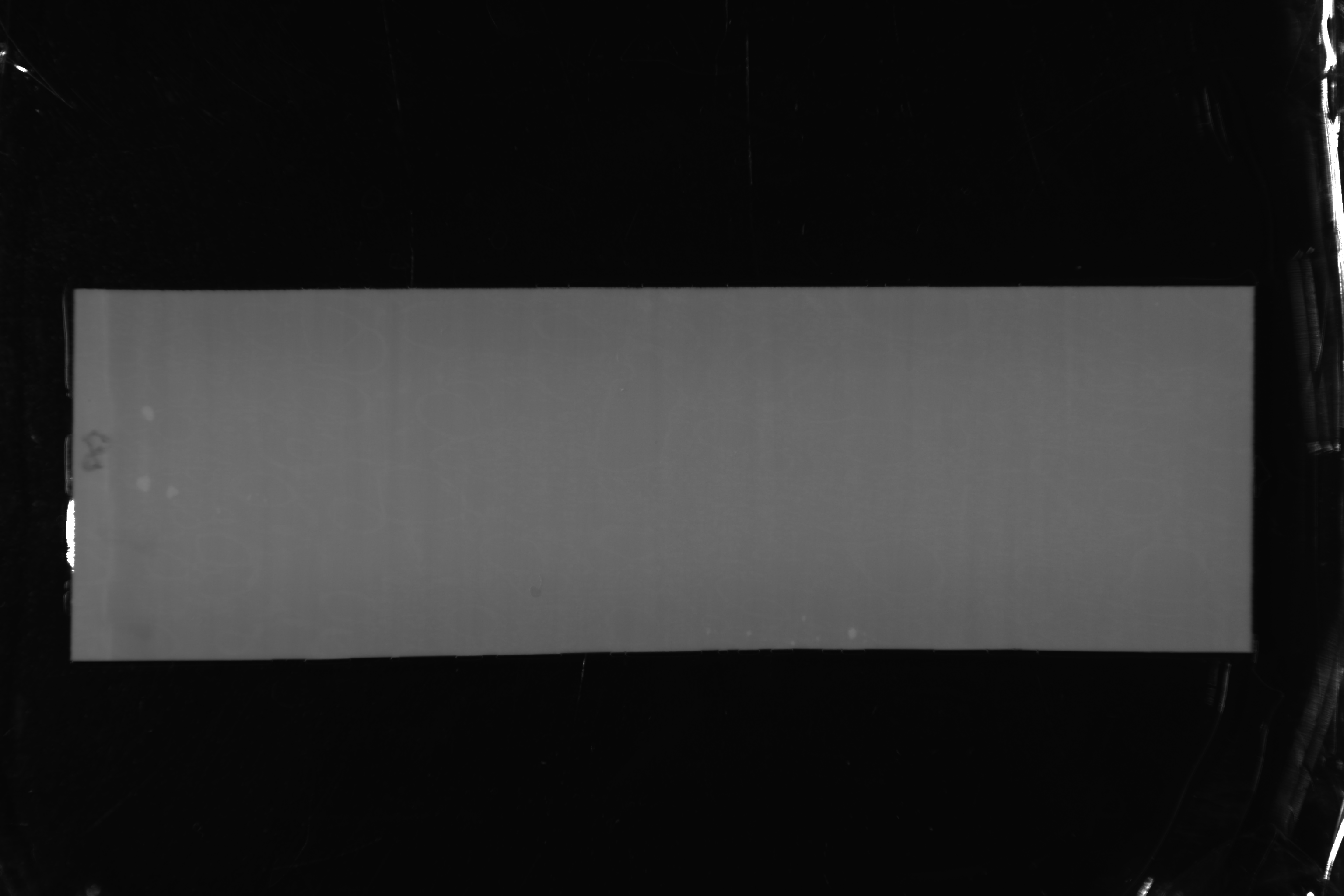

Supplement: Figure 5—source data 1. — Chemiluminescence and epi-illumination images of the blot membrane. [file elife-84860-fig5-data1.zip › Figure 5-source data 1/Figure 5-source data 1_epi-illumination.tif.tif]

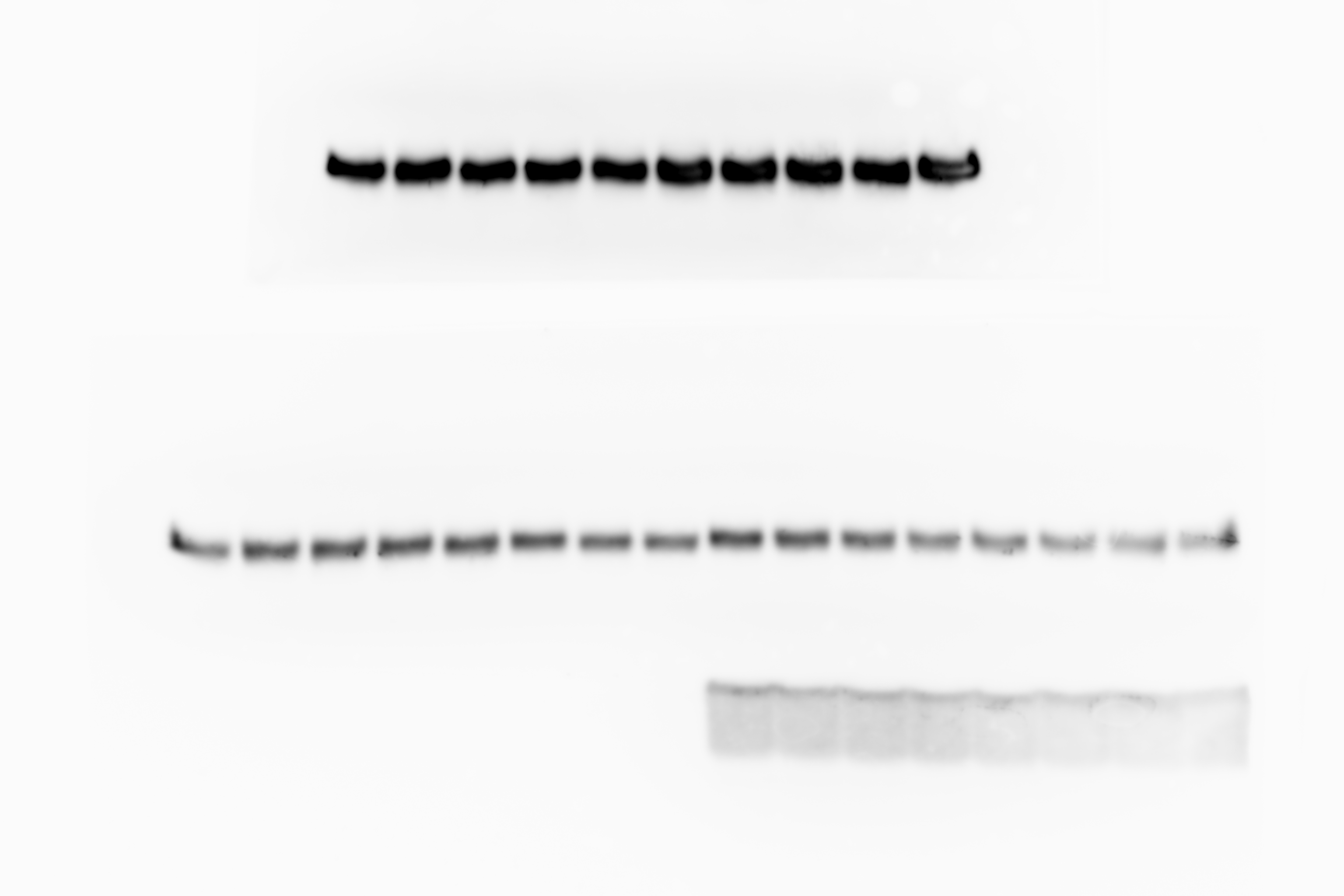

Supplement: Figure 5—source data 2. — Chemiluminescence and epi-illumination images of the blot membrane. [file elife-84860-fig5-data2.zip › Figure 5-source data 2/Figure 5-source data 2_chemiluminescence.tif]

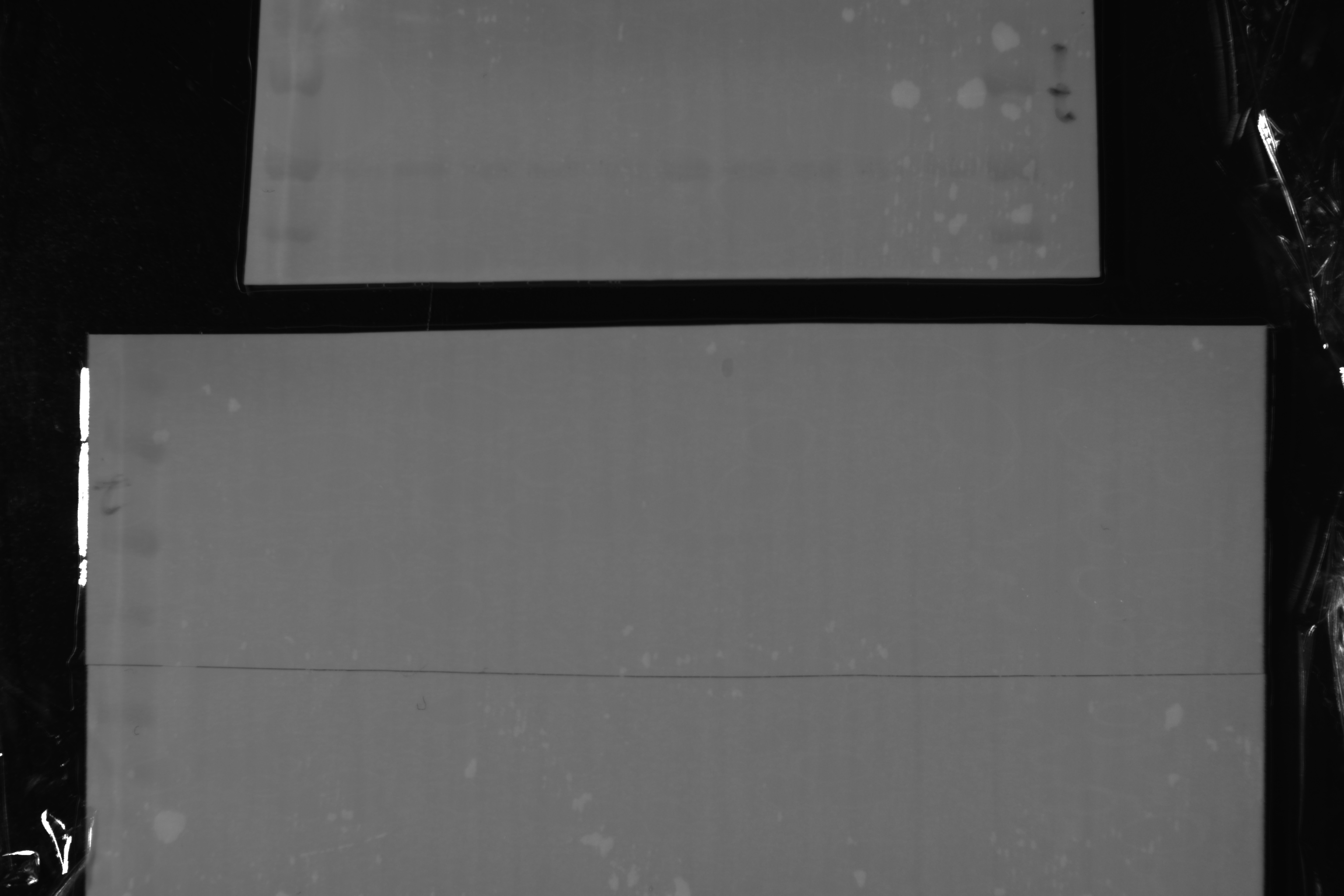

Supplement: Figure 5—source data 2. — Chemiluminescence and epi-illumination images of the blot membrane. [file elife-84860-fig5-data2.zip › Figure 5-source data 2/Figure 5-source data 2_epi-illumination.tif.tif]

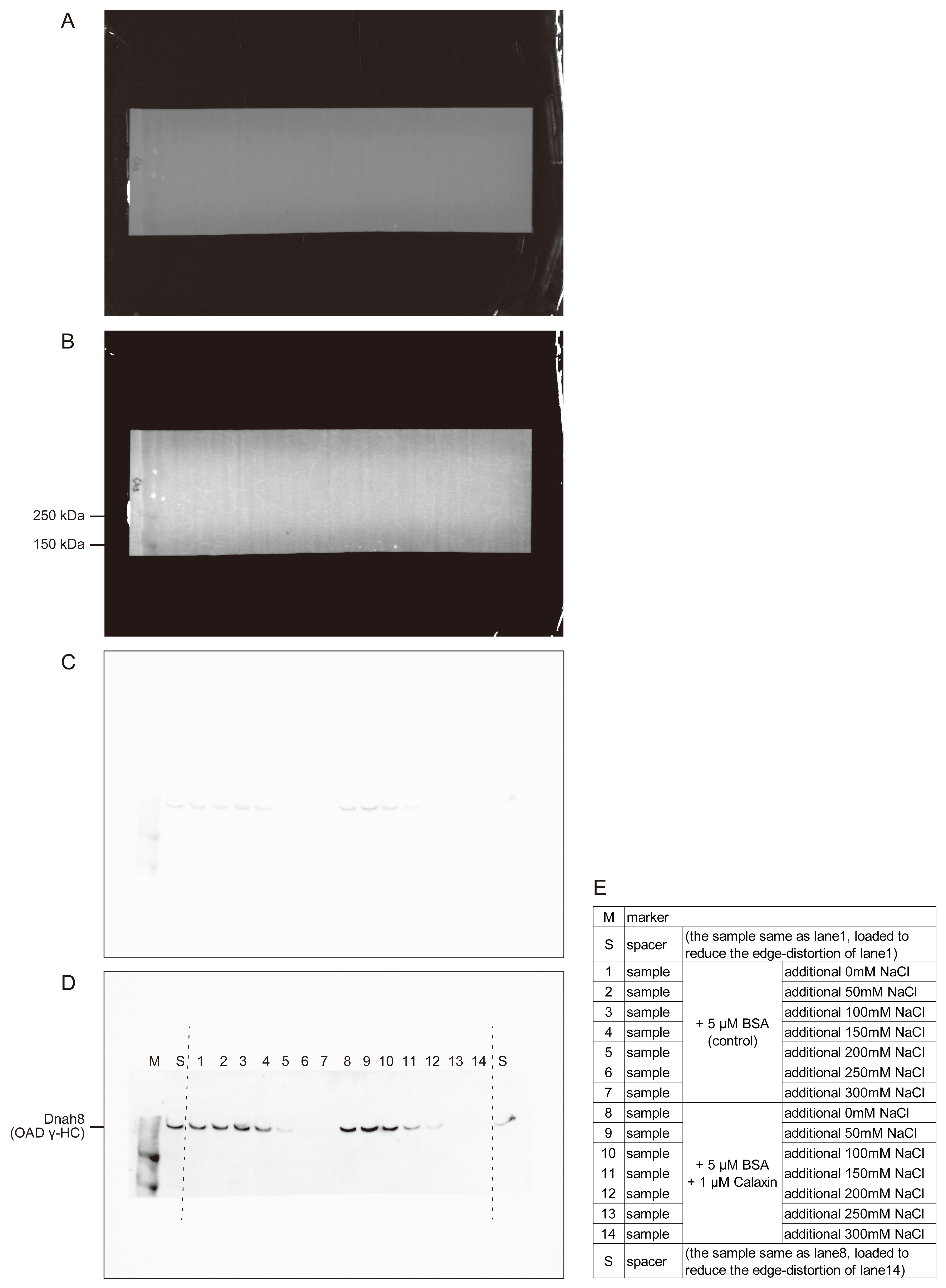

Supplement: Figure 5—source data 3. — (A) Original epi-illumination image. (B) Contrast adjusted image of A. (C) Original chemiluminescence image. (D) Contrast adjusted image of C, with annotations for each lane. (E) Annotations for lanes in D. [file elife-84860-fig5-data3.zip › Figure 5-source data 3/Figure 5-source data 3.jpg]

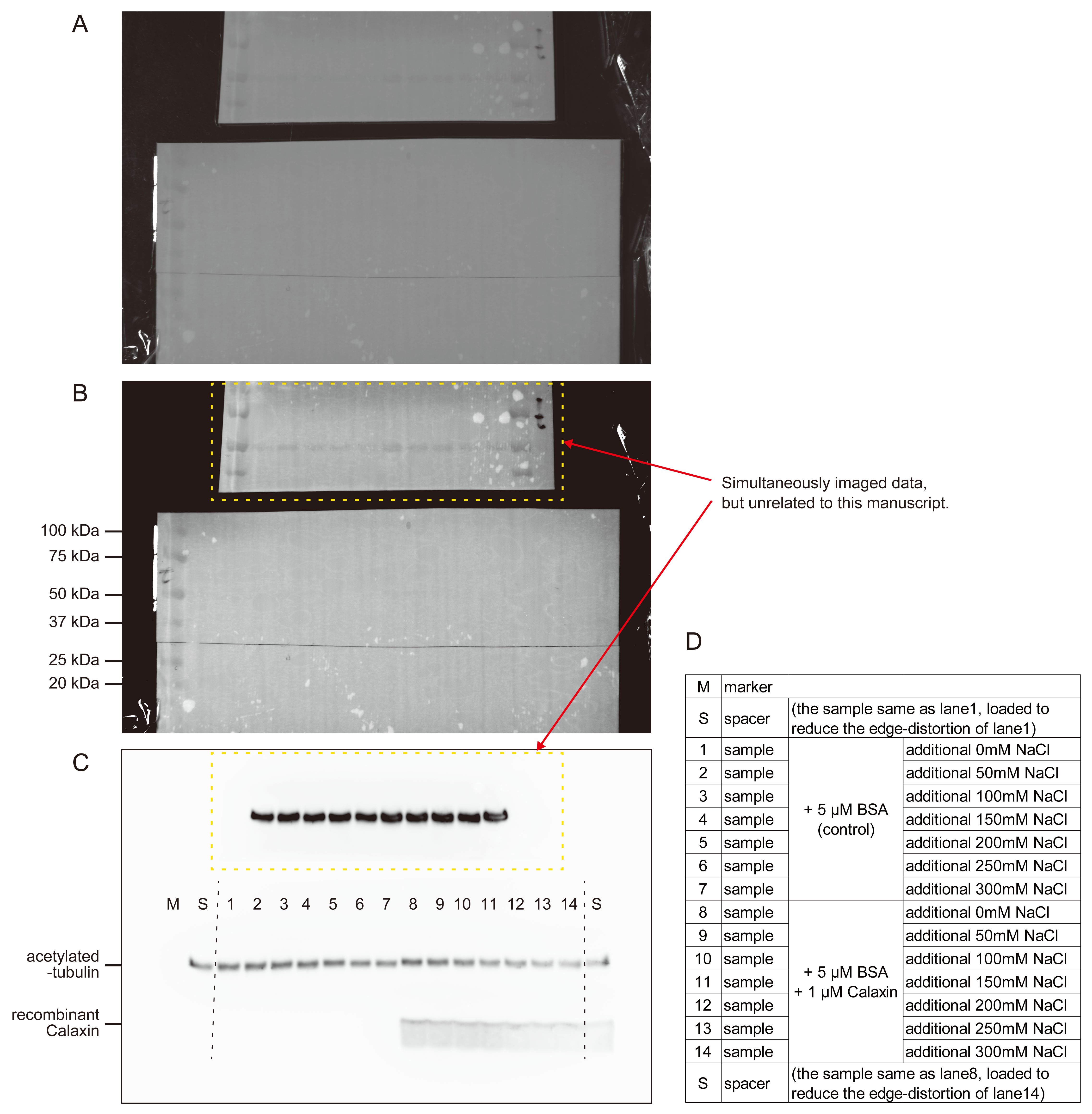

Supplement: Figure 5—source data 4. — (A) Original epi-illumination image. (B) Contrast adjusted image of A. (C) Original chemiluminescence image, with annotations for each lane. (D) Annotations for lanes in C. [file elife-84860-fig5-data4.zip › Figure 5-source data 4/Figure 5-source data 4.jpg]

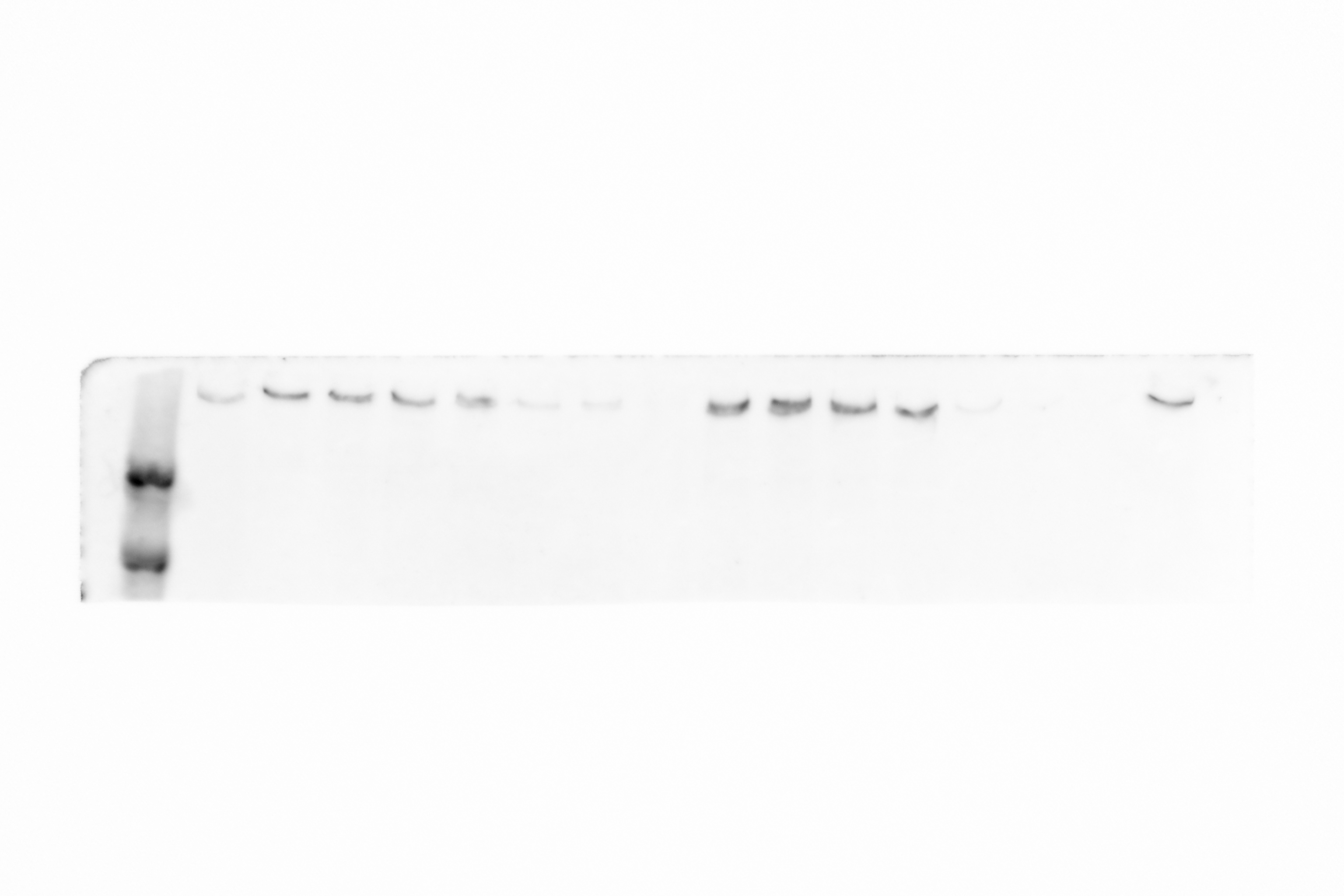

Supplement: Figure 5—figure supplement 2—source data 1. — Chemiluminescence and epi-illumination images of the blot membrane. [file elife-84860-fig5-figsupp2-data1.zip › Figure 5-figure supplement 2-source data 1/Figure 5-figure supplement 2-source data 1_chemiluminescence.tif]

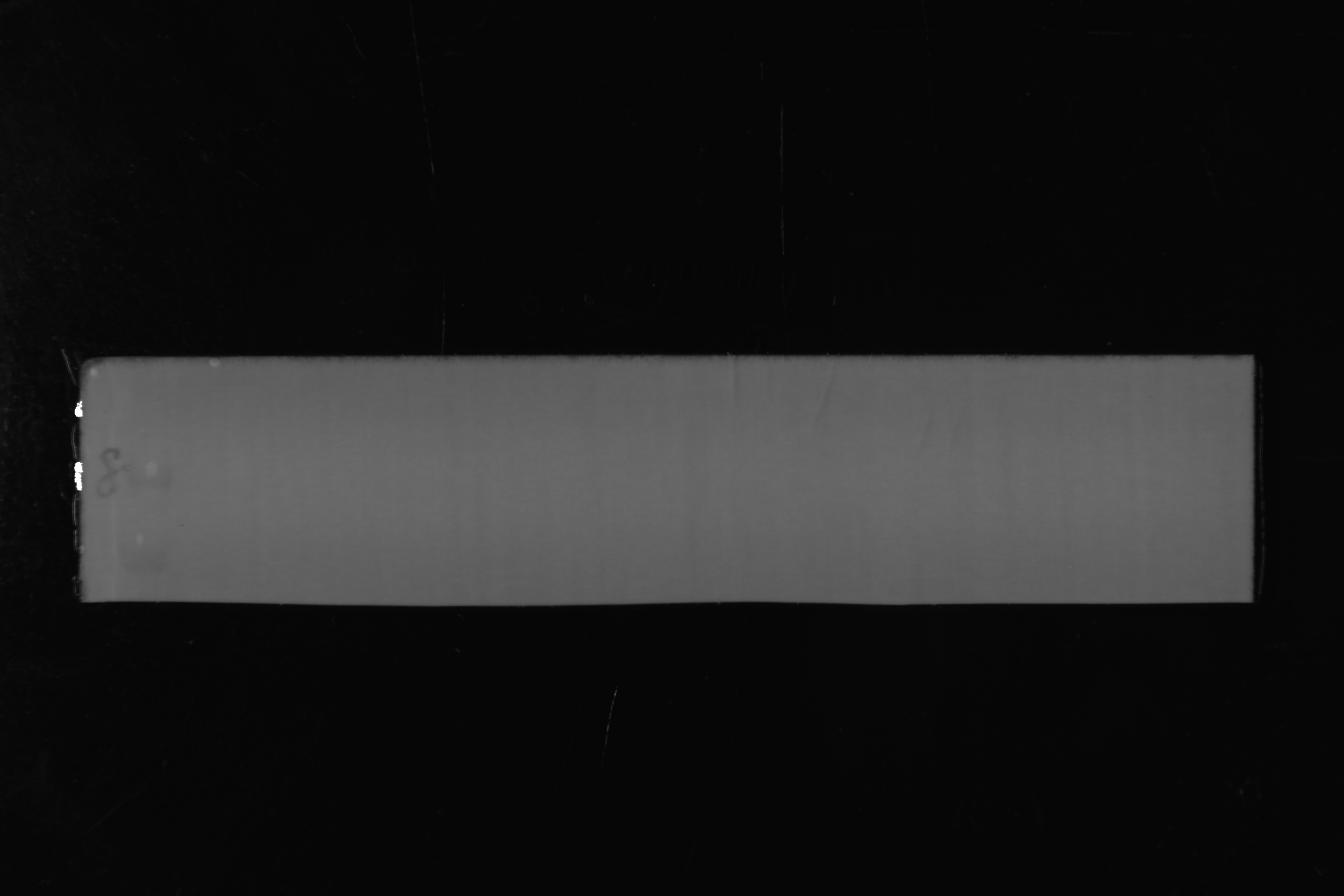

Supplement: Figure 5—figure supplement 2—source data 1. — Chemiluminescence and epi-illumination images of the blot membrane. [file elife-84860-fig5-figsupp2-data1.zip › Figure 5-figure supplement 2-source data 1/Figure 5-figure supplement 2-source data 1_epi-illumination.tif]

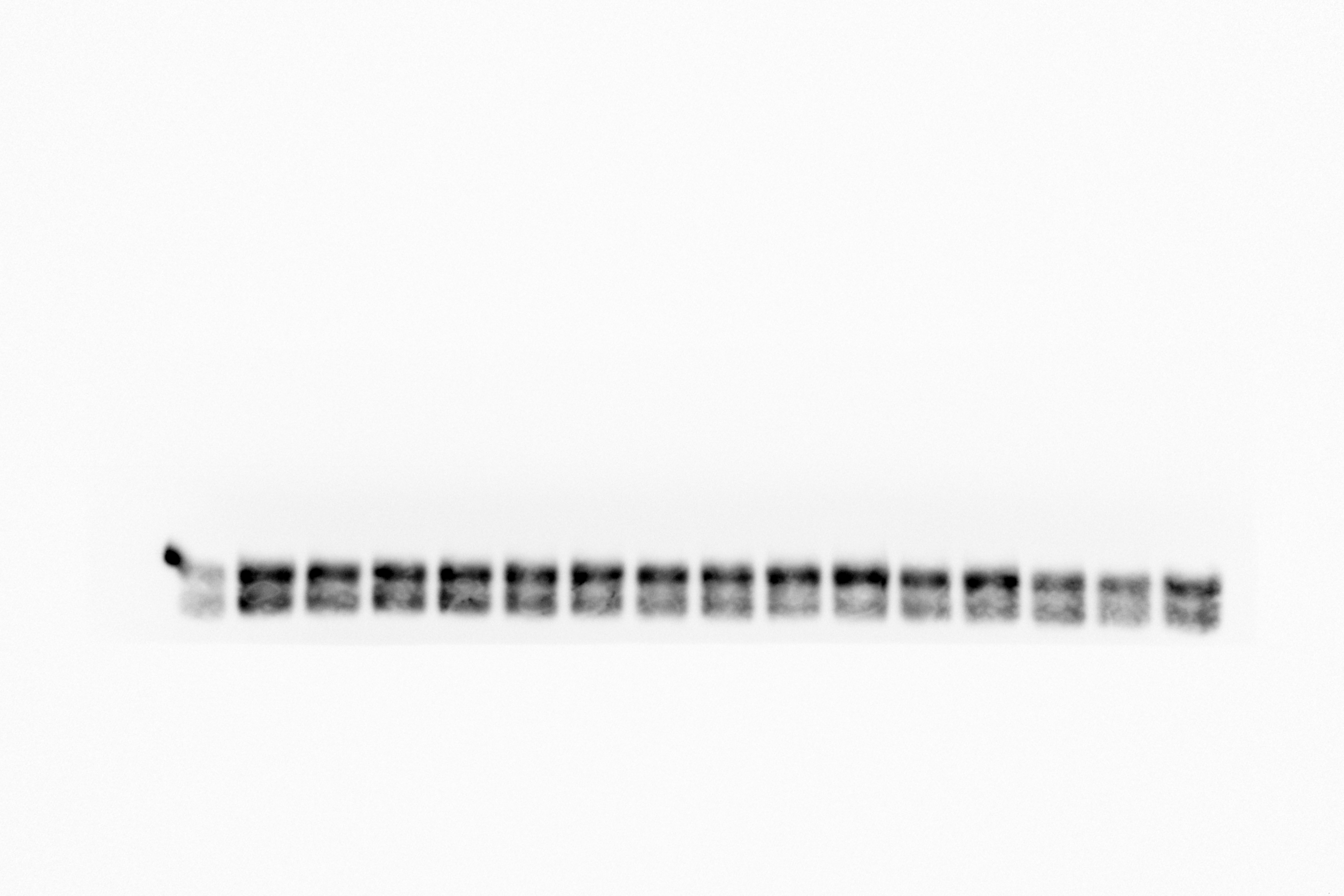

Supplement: Figure 5—figure supplement 2—source data 2. — Chemiluminescence and epi-illumination images of the blot membrane. [file elife-84860-fig5-figsupp2-data2.zip › Figure 5-figure supplement 2-source data 2/Figure 5-figure supplement 2-source data 2_chemiluminescence.tif]

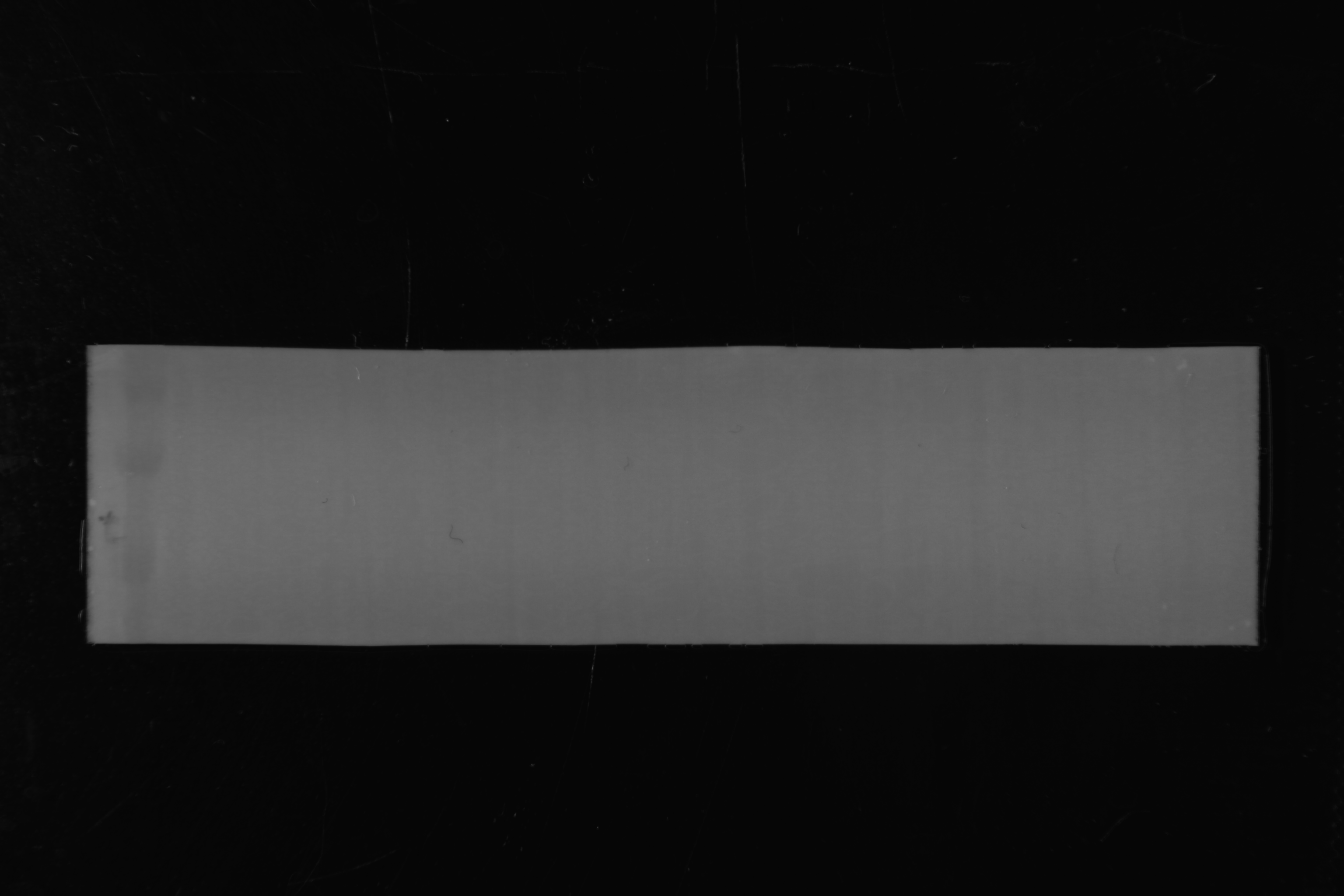

Supplement: Figure 5—figure supplement 2—source data 2. — Chemiluminescence and epi-illumination images of the blot membrane. [file elife-84860-fig5-figsupp2-data2.zip › Figure 5-figure supplement 2-source data 2/Figure 5-figure supplement 2-source data 2_epi-illumination.tif]

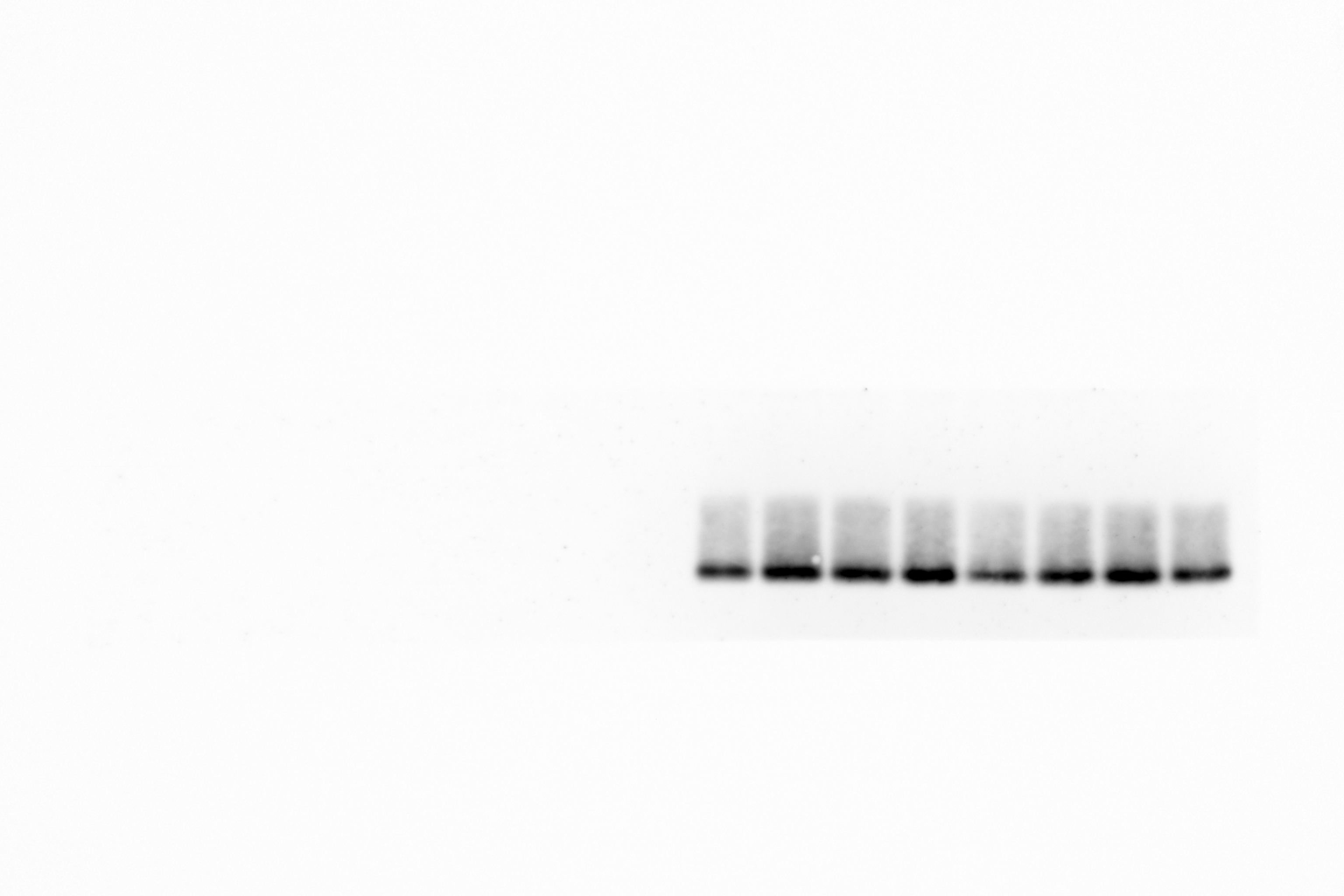

Supplement: Figure 5—figure supplement 2—source data 3. — Chemiluminescence and epi-illumination images of the blot membrane. [file elife-84860-fig5-figsupp2-data3.zip › Figure 5-figure supplement 2-source data 3/Figure 5-figure supplement 2-source data 3_chemiluminescence.tif]

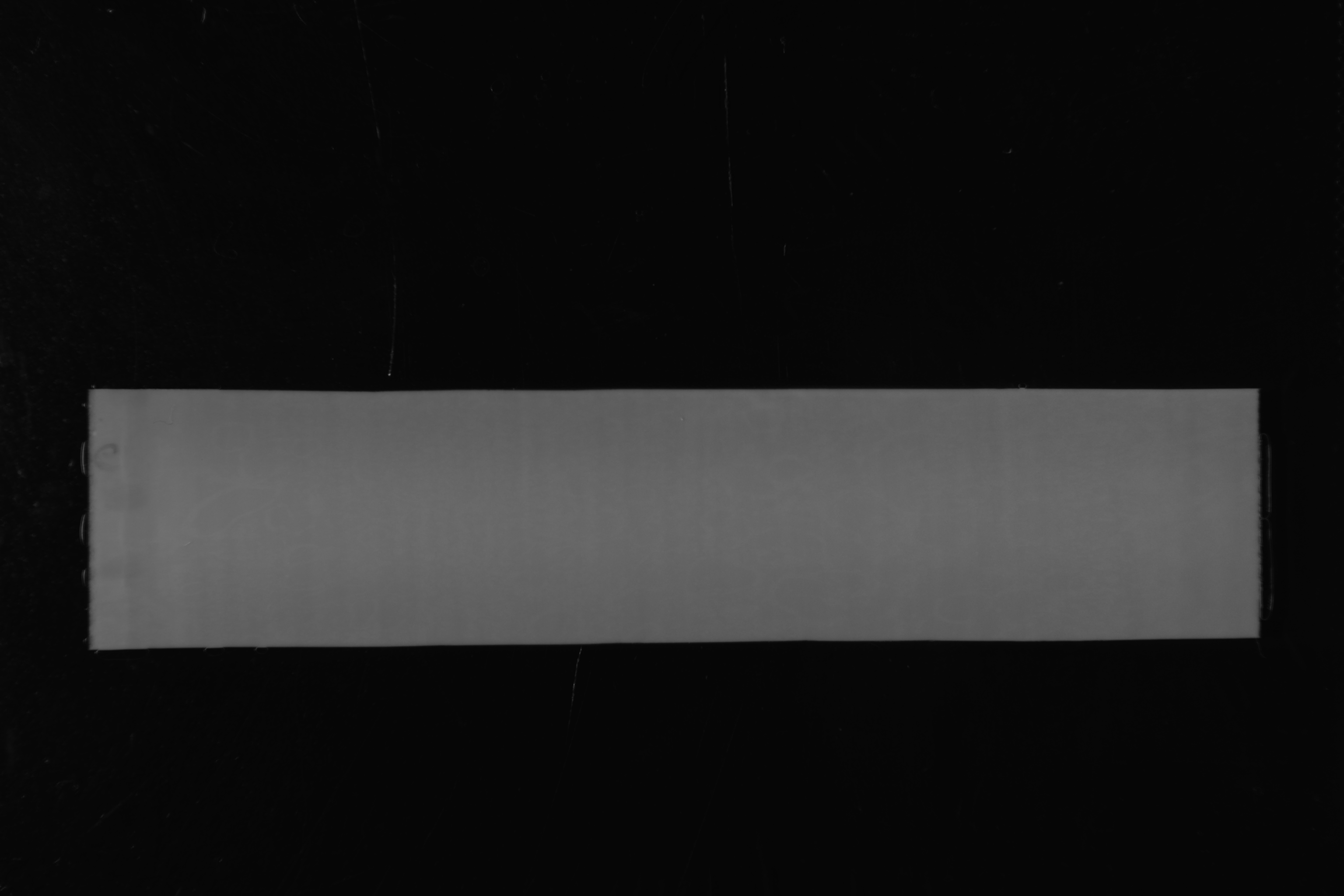

Supplement: Figure 5—figure supplement 2—source data 3. — Chemiluminescence and epi-illumination images of the blot membrane. [file elife-84860-fig5-figsupp2-data3.zip › Figure 5-figure supplement 2-source data 3/Figure 5-figure supplement 2-source data 3_epi-illumination.tif]

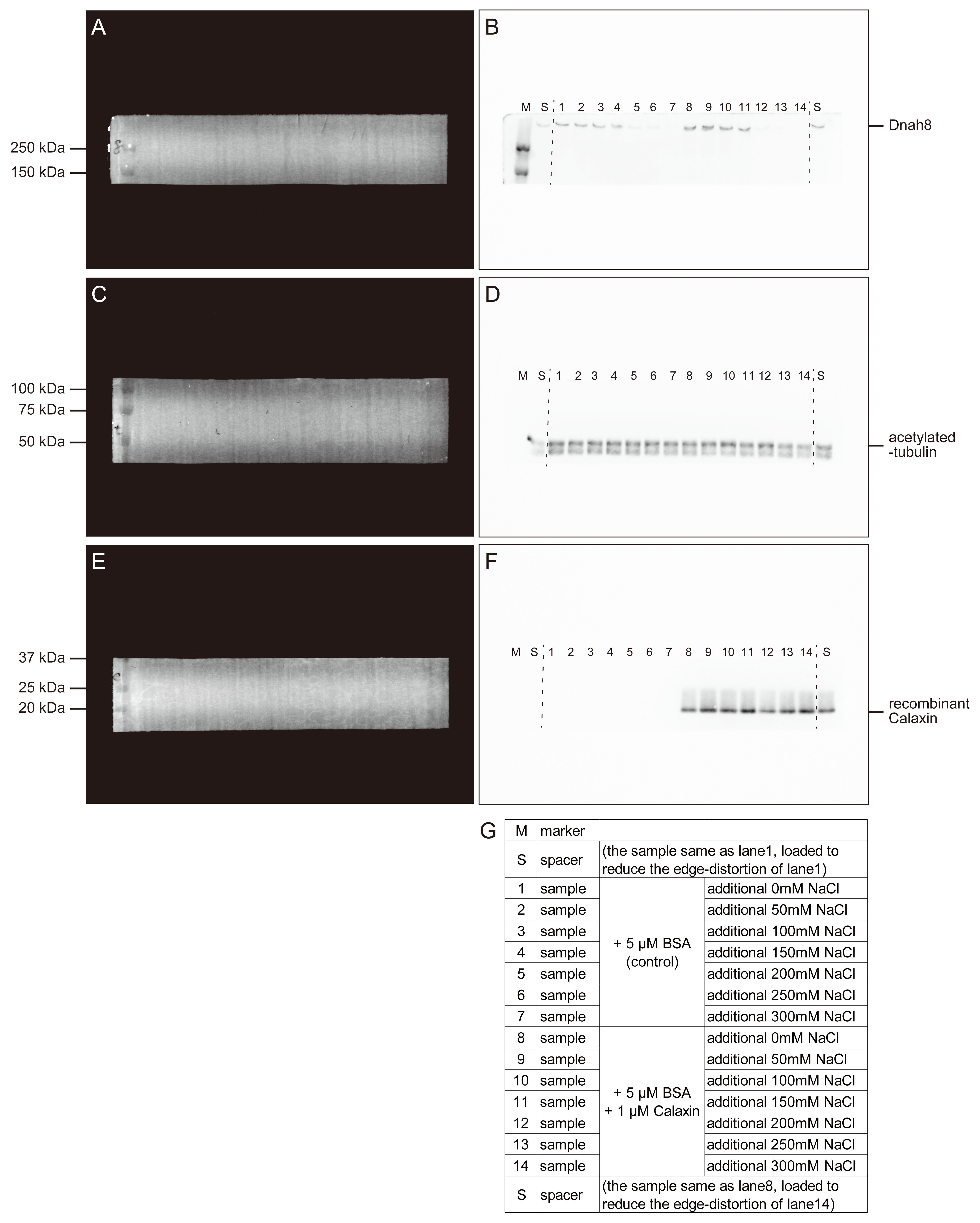

Supplement: Figure 5—figure supplement 2—source data 4. — (A, C, E) Epi-illumination images (contrast adjusted). (B, D, F) Chemiluminescence images (contrast adjusted), with annotations for each lane. (G) Annotations for lanes in B, D, and F. [file elife-84860-fig5-figsupp2-data4.zip › Figure 5-figure supplement 2-source data 4/Figure 5-figure supplement 2-source data 4.jpg]
